# Depression and Antidepressants: A Nordic Perspective

**DOI:** 10.3389/fpubh.2013.00030

**Published:** 2013-08-26

**Authors:** Andreas Vilhelmsson

**Affiliations:** ^1^Nordic School of Public Health, Gothenburg, Sweden

**Keywords:** depression, antidepressants, public health, Nordic countries, medicine

## A Nordic Welfare Model?

The Nordic countries are all established welfare states, and there has for some time existed a notion of a distinctive Nordic or Scandinavian welfare state; it is often understood in terms of broad, tax-financed public responsibility and legislated, collective, and universalistic solutions that respect employment interest yet aim at welfare and equity goals (1). Lately, the Nordic countries have performed well in comparative research of health policy in European countries (2) but also regarding health care system in OECD countries (3). However, while it appears that the case for the existence of a Nordic model is strong there is actually no consensus of the precise specification of the feature that defines the model (1). For example, considerable differences seem to exist between the psychiatric services within, as well as between, the Nordic countries when it comes to history, mental health acts and allocation of resources (4). Furthermore, some scholars argue that it is not even possible to speak of a common Nordic political approach to public health, since the public health programs in the different Nordic countries contain contradictory policies and ideological statements (5). This is especially evident for depression and sales of antidepressants in the different Nordic countries.

## Depression in the Nordic Countries

Depression is now regarded by the World Health Organization to be one of the most burdensome diseases in the world and deemed a public health priority (6). Overall, depression is estimated to have a point prevalence of about 5% in a general population, and a lifetime risk of about 15% (7). The prevalence of depression in the Nordic countries is believed to vary between 3.5 and 5% (8–12). However, information on depression prevalence in the different Nordic countries is quite difficult to find and the Nordic Governmental Websites does not seem to be up-to-date. For example, according to the Danish Centre for Health Technology Assessment (DACEHTA), the prevalence of depression in Denmark is not especially well investigated (13) and often relies on earlier research suggesting a point prevalence of 3% of the Danish population or 150 000 individuals (13, 14). More recent research has suggested that the prevalence of major depression disorder (MDD) in Denmark 2000–2006 increased from 2.0 to 4.9% and is now suggested to be of public health importance (10). The public health concerns is also raised in Finland and according to the Finnish Health 2000 project over 5% of the Finnish population seem to suffer from depression (9). Iceland is for some reason often not included in Nordic or European comparative studies and information on the prevalence of depression in Iceland seems to be scarce (15). According to information on their Website 15–25% of all Icelanders can expect to be depressed sometimes in their life and 12 000–15 000 individuals are predicted to suffer from depression at any given moment (10). This is equal to 3.8–4.8% of the Icelandic population. In Sweden, information provided by the Medical Products Agency suggests that approximately 5% of the Swedish population is afflicted by depression (12). In Norway, according to the classic Norwegian psychiatric epidemiological HUNT studies, depression affects approximately 3.5% of the Norwegian population (11).

## Use of Antidepressants

**Figure 1:**
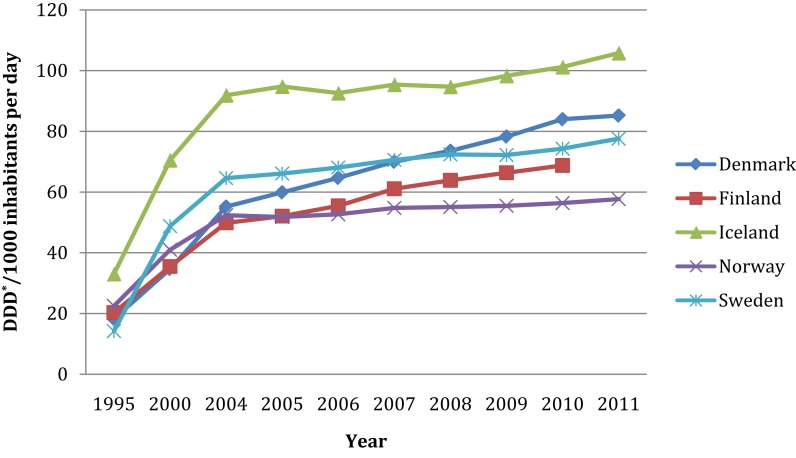
Sales of antidepressants (N06A) in the Nordic Countries during 1995–2011 in DDD*/1000 inhabitants per day. *Defined Daily Doses according to WHO classification.

The most common form of treatment of depression is antidepressant medication. They are currently ranked ninth among prescription drugs with global sales well over $20 billion (16). Overall, antidepressant prescriptions have risen, but this has been offset by a number of patent expiries and generic alternatives (17). As Figure 1 shows, sales of antidepressants in the Nordic countries have increased up to fourfold since the middle of the 90s (18, 19).

The overall sales of antidepressant drugs in the Nordic countries in 2009 (74.1 DDD/1000 inhabitants per day) is considerable higher than the OECD average (52.5), but also higher than for example in the UK (60.9) (20). However, there are great variations between the countries, where Iceland by far has the highest level of antidepressant sales, almost double that in Norway. In total, approximately 2 million Nordic inhabitants were prescribed an antidepressant, almost a share of 8.5% at a total cost of €236 million according to latest available statistics (ranging from 2010 to 2012). Danish medical statistics shows that there were sales of antidepressants in 2011 of €68 million and over 460 000 Danes were prescribed an antidepressant (8.3% of the population) (21). According to the Finnish Medicines Agency Fimea and Social Insurance Institution in 2010, estimated sales for antidepressants in Finland reached well over €44 million and more than 430 000 Finns were prescribed an antidepressant in 2010 (8.3% of the total Finnish population) (22). As reported by the Icelandic Medicines Agency estimated sales for antidepressants in 2010 were approximately €4 million (23), and over 35 000 patients in Iceland were prescribed an antidepressant in 2011 (11.2% of the total population) (24). Almost 300 000 Norwegians were prescribed an antidepressant in 2011 (6.3% of the population) and estimated sales were almost €50 million (25). In 2010, approximately 8.1% of the Swedish population did purchase an antidepressant drug and more than 5 million prescriptions of antidepressants were dispensed to almost 760 000 patients (26); antidepressant sales were estimated to almost €70 million (27).

## How Can This Rise in Antidepressants be Explained?

Several factors such as accessibility of drugs, available treatment alternatives, clinical practice and national guidelines, may influence patterns of prescribing and use of antidepressant drugs in the Nordic countries. Iceland has had a remarkable increase in antidepressant sales since 1995 (as indicated in Figure 1). An Icelandic study examining public views on antidepressant treatment suggested that the reason for the high usage of antidepressant in Iceland was a result of their perceived effectiveness by users, but also an effect of limited access to alternative treatment like psychotherapy (28). On the other hand, additional Icelandic research has suggested that despite an increase in antidepressants there were no positive impact on public health; instead the rates of psychiatric outpatient consultation and in patient treatment for depressive disorder increased, leading to increased medical costs (29). In a public health perspective this medical approach may seems questionable, but without further research on depression prevalence and why antidepressants are prescribed it is difficult to assess potential public health effects. This is of particular concern, since antidepressants in the absence of therapeutics alternatives are projected to continue to dominate the antidepressant market to 2018 (17).

Further research is therefore needed to scrutinize as to why differences in prevalence of depression and antidepressant sales exist between the Nordic countries, despite the Nordic model. The Nordic countries do for some reason have a high consumption of antidepressants compared to OECD despite relatively moderate or low depression prevalence patterns. This is especially important, since the increase in antidepressants consumption has spurred an ongoing debate whether antidepressants are overprescribed (30) (medicalization) or underprescribed (31) (poor access to treatment). With the increasing burden of disease due to mental disorders worldwide, knowledge of the epidemiology of these disorders are of increasing interest, and as indicated by others scholars (32) the Nordic countries have a strong history in this field of research. Otherwise valuable Nordic public health research may never be performed, despite its merits and its potential.

## Funding

This study has received funding from Letterstedska föreningen and Lundgrenska Fonden. The sponsor had no role in the study design; in the collection, analysis, and interpretation of data; in the writing of the manuscript; and in the decision to submit the article for publication. The researcher was independent of the funders.
